# A Smart Service System for Spatial Intelligence and Onboard Navigation for Individuals with Visual Impairment (VIS^4^ION Thailand): study protocol of a randomized controlled trial of visually impaired students at the Ratchasuda College, Thailand

**DOI:** 10.1186/s13063-023-07173-8

**Published:** 2023-03-07

**Authors:** Mahya Beheshti, Tahereh Naeimi, Todd E. Hudson, Chen Feng, Pattanasak Mongkolwat, Wachara Riewpaiboon, William Seiple, Rajesh Vedanthan, John-Ross Rizzo

**Affiliations:** 1 Department of Rehabilitation Medicine, NYU Langone Health, New York, NY USA; 2 Department of Mechanical and Aerospace Engineering, NYU Tandon School of Engineering, New York, NY USA; 3 Department of Neurology, NYU Langone Health, New York, NY USA; 4 Department of Biomedical Engineering, NYU Tandon School of Engineering, New York, NY USA; 5 Department of Information and Communication Technology, Mahidol University, Salaya, Thailand; 6 Ratchasuda College, Mahidol University, Salaya, Thailand; 7 Lighthouse Guild, New York, NY USA; 8 Department of Ophthalmology, NYU Langone Health, New York, NY USA; 9 Department of Population Health, NYU Langone Health, New York, NY USA; 10 Department of Medicine, NYU Langone Health, New York, NY USA

**Keywords:** Visual impairment, Blind/low vision, Low- and/or middle-income countries, Assistive technology, Accessibility, Navigation, Adaptive mobility device

## Abstract

**Background:**

Blind/low vision (BLV) severely limits information about our three-dimensional world, leading to poor spatial cognition and impaired navigation. BLV engenders mobility losses, debility, illness, and premature mortality. These mobility losses have been associated with unemployment and severe compromises in quality of life. VI not only eviscerates mobility and safety but also, creates barriers to inclusive higher education. Although true in almost every high-income country, these startling facts are even more severe in low- and middle-income countries, such as Thailand. We aim to use VIS^4^ION (Visually Impaired Smart Service System for Spatial Intelligence and Onboard Navigation), an advanced wearable technology, to enable real-time access to microservices, providing a potential solution to close this gap and deliver consistent and reliable access to critical spatial information needed for mobility and orientation during navigation.

**Methods:**

We are leveraging 3D reconstruction and semantic segmentation techniques to create a digital twin of the campus that houses Mahidol University’s disability college. We will do cross-over randomization, and two groups of randomized VI students will deploy this augmented platform in two phases: a passive phase, during which the wearable will only record location, and an active phase, in which end users receive orientation cueing during location recording. A group will perform the active phase first, then the passive, and the other group will experiment reciprocally. We will assess for acceptability, appropriateness, and feasibility, focusing on experiences with VIS^4^ION. In addition, we will test another cohort of students for navigational, health, and well-being improvements, comparing weeks 1 to 4. We will also conduct a process evaluation according to the Saunders Framework. Finally, we will extend our computer vision and digital twinning technique to a 12-block spatial grid in Bangkok, providing aid in a more complex environment.

**Discussion:**

Although electronic navigation aids seem like an attractive solution, there are several barriers to their use; chief among them is their dependence on either environmental (sensor-based) infrastructure or WiFi/cell “connectivity” infrastructure or both. These barriers limit their widespread adoption, particularly in low-and-middle-income countries. Here we propose a navigation solution that operates independently of both environmental and Wi-Fi/cell infrastructure. We predict the proposed platform supports spatial cognition in BLV populations, augmenting personal freedom and agency, and promoting health and well-being.

**Trial registration:**

ClinicalTrials.gov under the identifier: NCT03174314, Registered 2017.06.02.

## Administrative information

**Table:** 

Title {1}	A Smart Service System for Spatial Intelligence and Onboard Navigation for Individuals with Visual Impairment (VIS^4^ION Thailand): study protocol of a randomized controlled trial of visually impaired students at the Ratchasuda College, Thailand
Trial registration {2a and 2b}.	ClinicalTrials.gov identifier: NCT03174314
Protocol version {3}	2022.01.06
Funding {4}	Research reported in this publication was supported by the National Eye Institute of the National Institutes of Health under Award Number R21EY033689. The content is solely the responsibility of the authors and does not necessarily represent the official views of the National Institutes of Health.
Author details {5a}	1.Department of Rehabilitation Medicine, NYU Langone Health Mahya Beheshti, Tahereh Naeimi, Todd E. Hudson, John**-**Ross Rizzo 2.Department of Neurology, NYU Langone Health Todd E. Hudson, John**-**Ross Rizzo 3.Department of Biomedical Engineering, NYU Tandon School of Engineering Todd E. Hudson, John**-**Ross Rizzo 4.Department of Mechanical & Aerospace Engineering, NYU Tandon School of Engineering Chen Feng, John**-**Ross Rizzo, Mahya Beheshti 5.Department of Information and Communication Technology, Mahidol University Pattanasak Mongkolwat 6.Ratchasuda College, Mahidol University Wachara Riewpaiboon 7.Lighthouse Guild, New York, NY William Seiple 8.Department of Ophthalmology, NYU Langone Health William Seiple 9.Department of Population Health, NYU Langone Health Rajesh Vedanthan 10.Department of Medicine, NYU Langone Health Rajesh Vedanthan
Name and contact information for the trial sponsor {5b}	Jay Colbert Grant Specialist NATIONAL EYE INSTITUTE Jay.Colbert@NIH.GOV 301–451-4714
Role of sponsor {5c}	The sponsor takes on legal responsibility for the initiation, management and/or financing of the research
Composition, roles, and responsibilities of the coordinating centre, steering committee, endpoint adjudication committee, data management team, and other individuals or groups overseeing the trial, if applicable {5d}	N/A [no other groups overseeing the trial]

## Introduction

### Background and rationale {6a}

#### An international perspective on visual disability

Globally, there are 39 million blind and 246 million visually impaired people [1]. The economic impact of visual impairment was estimated to approach $3 trillion [2]. Assistive technologies enable people to be more productive, more independent, and achieve a higher quality of life. Despite the benefits derived from assistive technologies (AT), some parts of the world have minimal or no access to AT. In many low-income and middle-income countries (LMIC), only 5–15% of people who require AT have access to them. Rapid demographic changes will exacerbate this situation as populations over 60 years of age in LMIC are expected to be higher than in high-income countries in the coming years. Given both these trends, ATs are likely to be in high demand and help in our response to aging and disability.

According to Thailand’s National Statistical Office, visual disability is the third most common type of disability. Around 192,640 persons (or about 9%) have a visual disability who qualify for special rights, including 57,520 in the 15–59 age bracket, 2027 in the 6–14 range, and the remainder over 60. While therapeutic advances are in the “pipeline” for a handful of conditions, there are a multitude of causes that engender severe visual disability [3, 4], and the prevalence of many of these conditions is on the rise [5]. In addition to impaired mobility [6–8], vision impairments lead to unemployment [9], quality of life loss [10–12], and functional dependency [13], all of which reduce psychosocial well-being. More than 60% of visually impaired adults in Thailand face problems related to independent mobility, domestic activity, and performing activities of daily living [14]. Thai students with VI face barriers in day-to-day activities related to blind unfriendly physical structures, particularly those on educational campuses [15]. VI has also been associated with depression, decreased physical activity, impaired balance, and falls in Thailand [16, 17].

#### Increasing mobility: a persistent challenge

The concept of mobility is an essential aspect for all citizens. Given the development of cities, the complexity of successfully navigating between locations increases each day. Although there exists a potpourri of possible solutions to help promote mobility for the general citizen, there are particular design considerations for people with blindness and low vision.

Overall, mobility and wayfinding are difficult and often riddled with danger for people with BLV, especially in urban areas. The last century has seen the development of many accessibility devices and applications to assist BLV individuals, but few have been widely adopted; the white cane and guide dog are still the most commonly used mobility tools [18]. The use of a device is dependent on its characteristics, the application domain, and the user. In general, simple devices employed for use during seated tasks have the highest continued usage (60–80%) [19, 20], while those for augmentation of daily living activities and mobility have had much lower long-term usage rates (less than 20%) [21]. One possible explanation for the low acceptance of past efforts could be due to not sufficiently addressing users’ needs. As a result, most blind and low-vision students spend time after school alone without interacting with others [22]. In higher educational settings, many blind-unfriendly physical environments on campus have been documented but not addressed due to the challenging and expensive nature of the barriers, as a result, students are discouraged from active social participation as such environments seriously hamper their ability to join in lecture-based interactions and social gatherings [23]. These are disheartening facts, since many BLV live in cities with multiple universities and many social opportunities, and have the potential to engage with rehabilitation services, employment opportunities, and public transportation [24].

Since the Thailand Rehabilitation Act of 1991, medical rehabilitation services for people with disabilities are increasingly available around the country. The Rehabilitation Act was subsequently renamed the Persons with Disability Empowerment Act in 2007 and orientation and mobility services are included within a universal health benefits package, along with white canes. However, while there has been a push in Thailand for the digitalization of society, there has not been any research and development on digital assistive technologies to improve mobility.

#### Wearable solutions: a paradigm shift

In our current era of self-driving vehicles, there is a clear need to move beyond the traditional approaches, such as the white cane, used in conjunction with a mélange of limited-use gadgets and apps. We propose a paradigm shift focused on comprehensive tools to make safe mobility a reality for the BLV. The long-term goal of our research team is to improve the quality of life of individuals who are adapting to low vision and blindness by providing connected, human-in-the-loop wearable solutions that foster true functional independence.

#### Spatial intelligence: user-centered design to improve outcomes

VIS^4^ION, a mobile computing platform capable of real-time scene understanding with human-in-the-loop navigation assistance, has four components: (1) a wearable**/**instrumented backpack with several distinct distances and ranging/image sensors to extract pertinent information about obstacles and the environment; (2) an embedded system (Edge AI-computer) with both computing and mobile (4G, 5G, and Wi-Fi) communication capability (*inside backpack*); (3) a haptic interface (waist strap) that communicates the spatial information computed from the sensory data to the end-user in real-time via an intuitive, torso-based, ergonomic, and personalized vibrotactile scheme; and (4) a headset that contains both binaural bone conduction speakers and a noise-canceling microphone for oral communication [25, 26]. The belt and headset are used to alert the end user of environmental features. A stereo camera is our main image sensor and acts as a sensing foundation. Advanced computer vision algorithms are deployed on the Nvidia Jetson Xavier processor board for continuous analysis of a dynamically changing environment [25, 26].

**Fig. 1:**
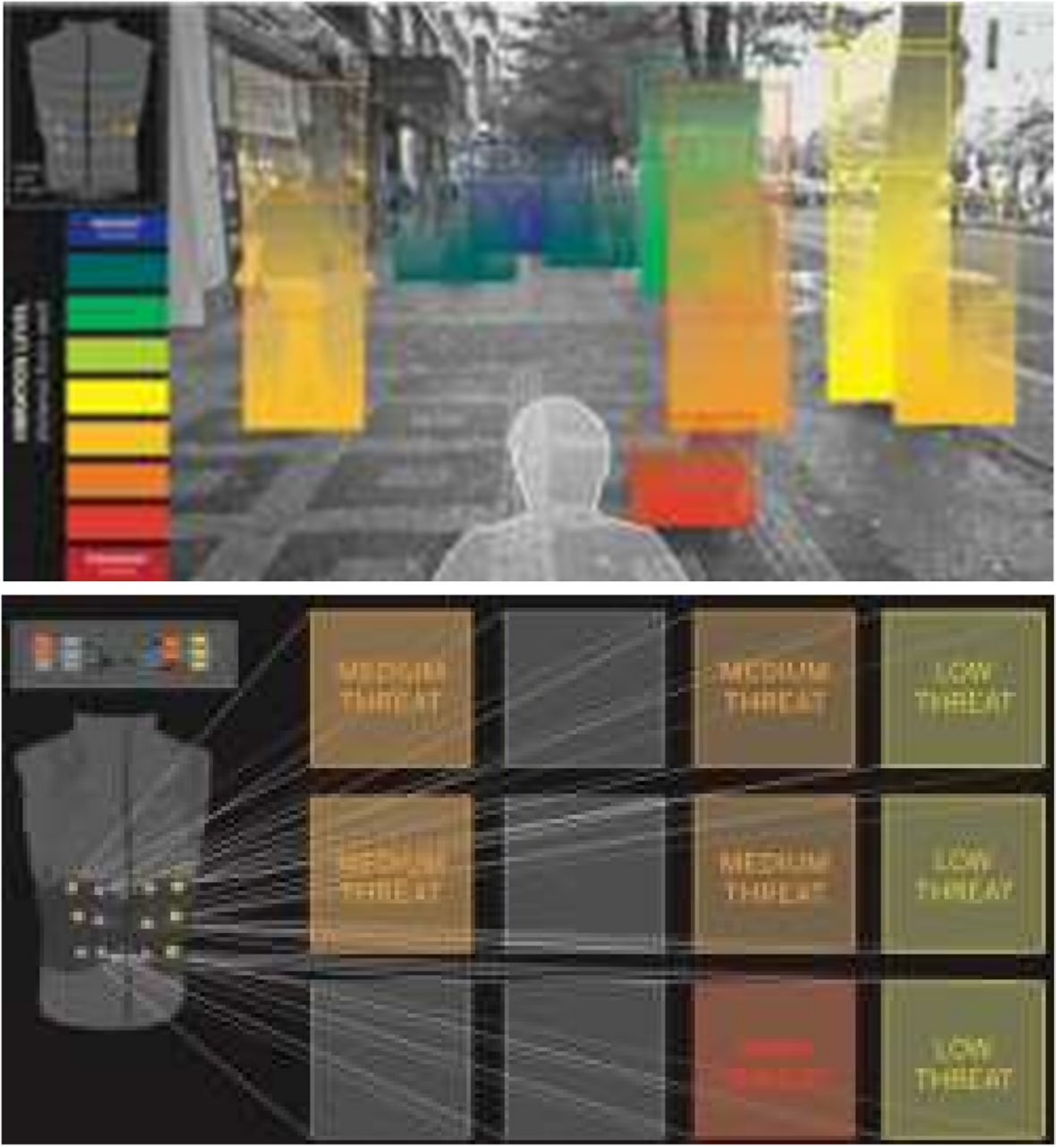
(Top) a simulated view of a scene decomposed into capture fields that spatiotopically correspond to actuators in the haptic interface; (bottom) color-coded depiction demonstrating the scene decomposed into a segmented grid for belt-based vibratory warnings of various threat levels based on proximity and spatial position (note: 12 tactors correspond to 12 capture fields)

The platform delivers a series of microservices to the end user. One basic feature is hazard-mapping. The scene, as viewed by the stereo camera, is decomposed into capture fields that correspond to the density of the tactors on the haptic interface in a spatiotopically preserved, intuitive, body-centered (egocentric) fashion (Fig. 1). Shorter hazards vibrate fewer (lower) actuator columns on the belt and obstacles that are closer to the user induce a higher frequency of vibration, giving a sense of tactile looming or “approach.”

With its user-friendly form factor, the platform is easily adoptable by BLV individuals. Yet, there is a pressing need to expand its function by refining its mapping/localization microservices and steering its development toward a defined social problem to fill the following gaps: (1) limited ability to interpret real-world scenes through computer vision algorithms and excessive computational burden (mapping); (2) lack of a scientifically-principled, experimentally-based understanding of users’ needs and behavioral patterns in complex settings, such as Thailand; (3) poor understanding of the implementation methods required to deploy and sustain such mapping and localization methods at scale effectively; and (4) lack of hypotheses-driven studies with ecological validity.

### Explanation for choice of comparators {6b}

This study aims to test the hypothesis that VIS^4^ION is a wearable assistive technology that assists spatial cognition in VI populations, enhancing personal freedom and independence, health, and well-being. VIS^4^ION is able to take advantage of contemporary, state-of-the-art technologies and approaches. The backpack embodiment was a careful decision made with end users to enhance acceptance; user-centric design approaches will be used for the entirety of the project.

There is a clear lack of comparison of the effect of wearable assistive technology on health and quality of life, including independent and social engagement in active and passive mode; trials evaluating the effectiveness of assistive technology rarely included mobility metrics, subjective and objective assessments of the quality of life, acceptability and appropriateness as comparators. Therefore, we will have comprehensive metrics to evaluate the outcomes in the first week and fourth week of extended use of wearables in passive and active phases. In “the passive phase,” the wearable will be maintained in a monitoring mode and simply acquire data, providing no assistance/feedback on location and orientation. During the active phase, the wearable will have an activated assistive mode, i.e., the wearable will acquire data and simultaneously provide assistance/audio feedback.

### Objectives {7}

The central hypothesis of our program is that the proposed assistive wearable will support spatial cognition and more efficient navigation, augmenting personal freedom and agency, and promoting health and well-being in people with BLV. In order to achieve this goal, we will complete the following objectives:

1. Digital twin (map) a campus in Thailand to enable accurate and precise localization for students on campus**.** Therefore, a more efficient navigation support service will be created and foster optimal wayfinding. We will test mobility and anticipate greater activity and a wider home range (more land coverage)**,** and shorter traversal times for given initial/intended map-points.
2. Test acceptability, appropriateness, and feasibility of our approach. We will adapt VIS^4^ION based on the input from this formative testing.
3. Perform an extended-use randomized controlled trial with VIS^4^ION.
4. Test a wider coverage area, assessing generalizability. We intend to connect our campus of interest to urban Bangkok.

### Trial design {8}

#### Phase 1

Aim 1: Create a “digital twin” of a particular campus (Ratchasuda — a disability college), testing both the performance of the service itself as well as the functional impact of the system.

Aim 2: Assess the ecology of VI students using the wearable, assessing the home range and activity level. A cohort of 20 VI students will use the wearable during navigation (1 week) in two phases: an initial passive phase, during which the wearable will only record location, and then an active phase, during which the location recording will be enhanced with orientation cueing.

Aim 3: Assess the acceptability, appropriateness, and feasibility of the wearable, as a navigation aid for the BLV, using the results of interviews and surveys to implement improvements to the system.

#### Phase 2

Aim 4: We designed an exploratory experiment to assess and monitor our participant’s health-related changes (increased mobility and QoL). This phase is planned as a prospective, randomized crossover, controlled, non-blinded study to compare to evaluate the effectiveness of the wearable for increasing navigation and quality of life. Twenty BLV participants will be randomized to group A (10 participants) and group B (10 participants). During the first 15 days, group A will go through an active phase and then a passive phase for the next 15 days. Reciprocally, group B will go through a passive phase for the first 15 days and then an active phase for the second half of the month.

Aim 5: We will test the generalizability of the new mapping service in a more challenging environment — testing system performance in Bangkok.

## Methods: participants, interventions, and outcomes

### Study setting {9}

Our study will be performed at Mahidol University in Thailand. Areas like this small town in Thailand lack sophisticated technologies and facilities and are therefore an important test case for evaluating a new technology that is intended to be used worldwide for BLV individuals in both low- and higher-income areas and countries.

To provide inclusive higher education for all and equal access to learning and socially engaging activities, Ratchasuda College at Salaya (the largest campus of Mahidol University) provides short course trainings on orientation and mobility, accessible information access for the blind, as well as master-level degree programs focused on blind rehabilitation.

As Ratchasuda College is aware of the bottlenecks in the education and employment of BLV students, pre-college activities have been created with eleven blind schools located throughout Thailand. Transition management and disability support services are mechanisms to facilitate progression to higher levels of education and employment. We aim to expand our scope using this network and these mechanisms; we also plan to leverage collaborations with institutions in the provinces of Khon Kaen and Songkla.

### Eligibility criteria {10}

Inclusion criteria (patients with visual impairment).

Participants with visual impairments will meet the following criteria:

1. Older than 18 years,
2. Medically diagnosed with visual impairment (legal blindness — as per the US government standards and the Social Security Administration, including acuity and/or field deficits)
3. Currently using a primary assistive device for mobility (e.g., white cane or guide dog)
4. Able to travel independently (without aid of another person)

Exclusion criteria include:

1. Significant injury to the upper and/or lower extremity,
2. Comorbid neurological illness,
3. Confounding medical conditions
4. Pregnancy
5. Auditory impairments
6. Somatosensory impairments to the trunk or torso that precludes the use of the haptic interfaces

### Who will take informed consent? {26a}

The consent process will take place in a private room by an IRB-approved study staff member. Once an appointment is scheduled with a participant who verbally expresses an interest in the study, a trained member of the research team will verbally explain the purpose of the study to the participants, the potential benefits and risks (minimal in this case) they may experience, and the voluntary nature of their participation. The language that will be used regarding the explanation of the study and participation consent will be no higher than a 6th grade language level and it will be in a language understandable to the subject (English\Thai). Written informed consent will be obtained when participants agree to participate, and they are able to explain their understanding of the study to the trained research team members. If they cannot, the research team member will review the study with them again, if they still cannot explain the study back, they will not be enrolled. A copy of the consent agreement will be provided to participants for reference. Any questions will also be answered at this time. All efforts will be made to make the consent process free of coercion.

### Additional consent provisions for collection and use of participant data and biological specimens {26b}

Not applicable, we will not collect and use participant data and biological specimens for ancillary studies.

### Intervention description {11a}

#### Aim 1

**Fig. 2:**
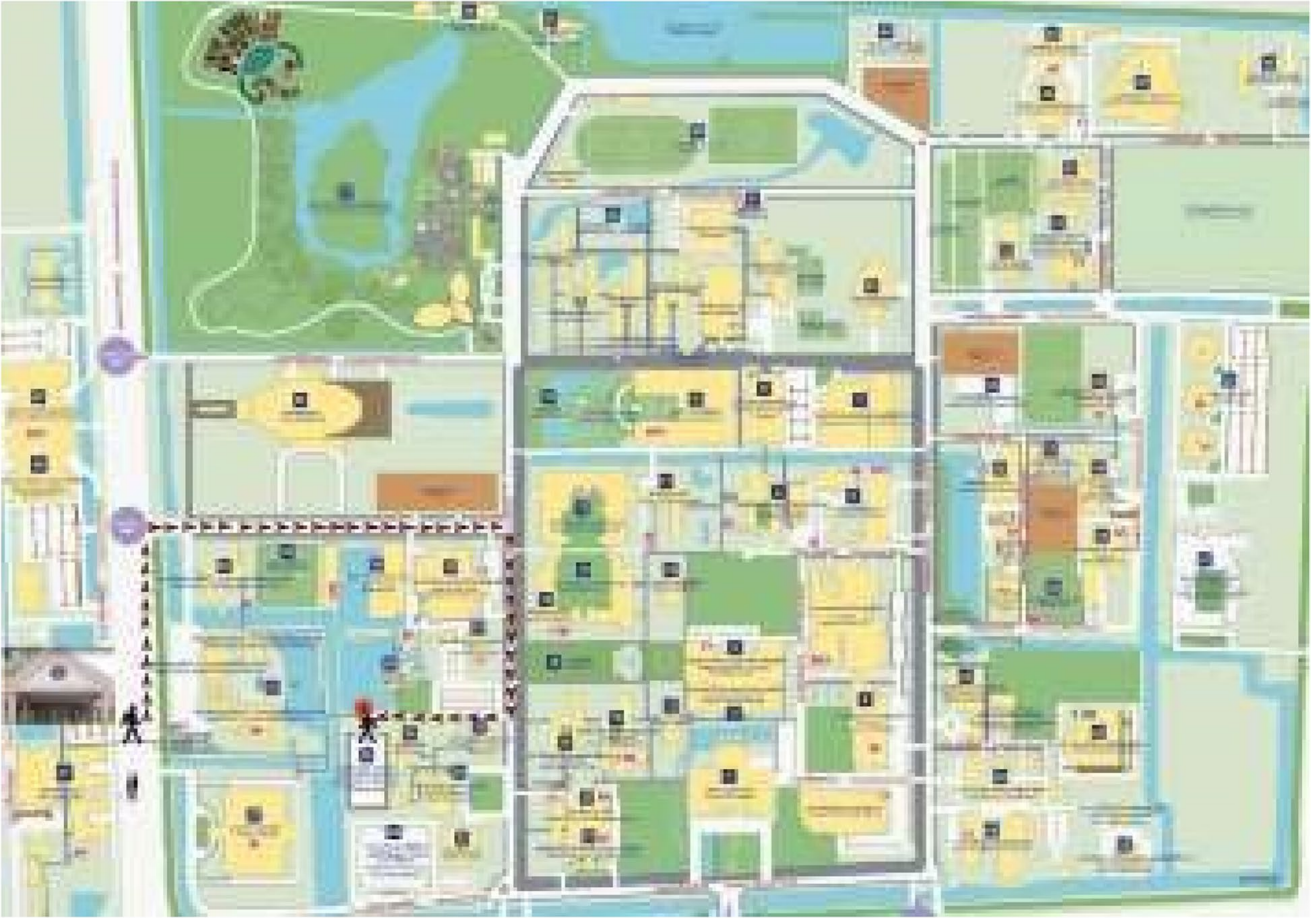
Aerial map of Ratchasuda College (RC) at Mahidol University. A route between two on-campus locations is delineated

Two study members will carry a specialized wearable for mapping with 3 extra stereoscopic camera units and physically ambulate the entire MU-RC campus, as pictured in Fig. 2. This data acquisition phase will be completed in a series of five 2-h blocks, as determined by a prior feasibility analysis. Each study member will complete two data acquisition recording series (i.e., 10 blocks). The mapping data will be compared intra-recorder (between series) and inter-recorder and then barring fidelity issues will be used for the pipeline, as delineated in the overview section. Each block will be pre-defined and reviewed with the study team member, at outset, and again on each recording day, and all data will be checked for validity. Following data acquisition and the mapping sequence (reconstructions), localization queries will be performed across RC (50 in total, evenly spread across campus) and compared to ground truth. We have defined parameters for city blocks covered/pedestrian hour of mapping and storage estimates for data acquired.

#### Aim 2

We will recruit a cohort of twenty BLV students currently enrolled in classes at Mahidol University. Students will be asked to don a wearable (bookbag) during all tasks requiring navigation for one week (10 days)**.** Ethograms will be constructed by coding the basic behaviors documented while navigating particular sections of campus, including public transport access points, street crossings, street hazards, e.g., areas with scaffolding, navigation to and through entrances, straight paths (to assess veering), curved paths, and elevation changes, e.g., stairs and ramps. We will collect data on the behavioral components of each situation to understand the effects of the environment on the participant as one moves through campus and the variables that trigger and modify related behaviors [27]. We will describe the mobility phenotype as BLV users interact with a complex range of obstacles in the various areas of campus, cataloging the vastly different reactions and consequences triggered by each. Particular obstacles and campus areas may have a higher likelihood of precipitating mechanical falls, leading to potentially catastrophic injuries. Thus, this process will guide the training of machine learning algorithms based on the specific task to be completed. For example, an intersection crossing near a bus stop for someone with a high degree of veering is particularly precarious; this and other related findings require specific attention and additional safety prompting. We will test navigation assistance (short-term) by comparing the initial five days with the VIS^4^ION system worn in passive mode (acquiring data, providing no assistance/feedback on location and orientation) to the final 5 days with the system worn in active or assistive mode (acquiring data and simultaneously providing assistance/audio feedback).

#### Aim 3

We will ask the same cohort of BLV students enrolled in Aim 2 to participate in focus group discussions (FGD) to assess the acceptability and appropriateness of VIS^4^ION. A research team member will conduct FGDs, each of which will consist of 6**–**8 individuals, and an interpreter will help facilitate the discussion [28].

Each FGD session will last roughly 60 min and include participants’ perceptions and experiences while navigating the VIS^4^ION system. The FGDs will be guided by pre-determined questions related to general perceptions of the VIS^4^ION system, as well as thoughts on the facilitators and barriers of the intervention. The FGD sessions will be audio-recorded verbatim and later transcribed and translated into English by the research team. During the sessions, the research team member will also take field notes.

#### Aim 4

A cohort of 20 (new) participants will be recruited to assess extended-use benefits over one month. We will create two groups for the randomized crossover design. Each participant will be randomized to either sequence A (10 participants) or sequence B (10 participants). During the first 15 days, group A will go through an active phase and then a passive phase for the next 15 days. Reciprocally, group B will go through a passive phase for the first 15 days and then an active phase for the second half of the month. During the passive phase, the wearable will be maintained in a monitoring mode and simply acquire data, providing no assistance/feedback on location and orientation. During the active phase, the wearable will have an activated assistive mode, i.e., the wearable will acquire data and simultaneously provide assistance/audio feedback. We will evaluate the effectiveness of the wearable for increasing navigation and quality of life. We hypothesize that the VIS^4^ION wearable will improve navigation and quality of life in individuals with visual disabilities.

During the active phase, subjects will complete their daily activities while wearing VIS^4^ION and using the mapping and localization microservice to facilitate wayfinding. Throughout the study we will monitor navigation performance through the same metrics as delineated in Aim 2 [week 2 compared to week 4], as well as to monitor for any falls that may occur (IMU-based alerting); we will also subjectively assess visual function through the 25-Item National Eye Institute Visual Function Questionnaire (VFQ25) [29], physical function and mobility, through the International Physical Activity Questionnaire (IPAQ) [30, 31], and QoL, through the World Health Organization QoL Brief (WHO-QoL-BREF) [32, 33]. The subjective assessments will be performed on day 0 (day of device delivery and initiation) and on the final day of the trial (day 31).

We will also use the Saunders Framework [34] to conduct a process evaluation of the intervention. This process evaluation will monitor program implementation to better understand the relationship between intervention components, process/implementation measures, and health outcomes. The Saunders Framework provides a systematic approach for assessing key process measures across several domains. This approach will generate both formative and summative data [35–37]. A combination of quantitative and qualitative approaches will be utilized, including observation forms, checklists, surveys, record review, FGDs, and KIIs (key informant interviews). Study participants, clinical staff, and study and clinical encounter forms will be the sources of data. Members of the research team will conduct semi-structured interviews with students’ post-experiment; the session will be audio-recorded verbatim. Interview audio-recordings will be transcribed and translated into English by the research team. Special emphasis will be placed on Thai cultural and lifestyle preferences.

#### Aim 5

**Fig. 3:**
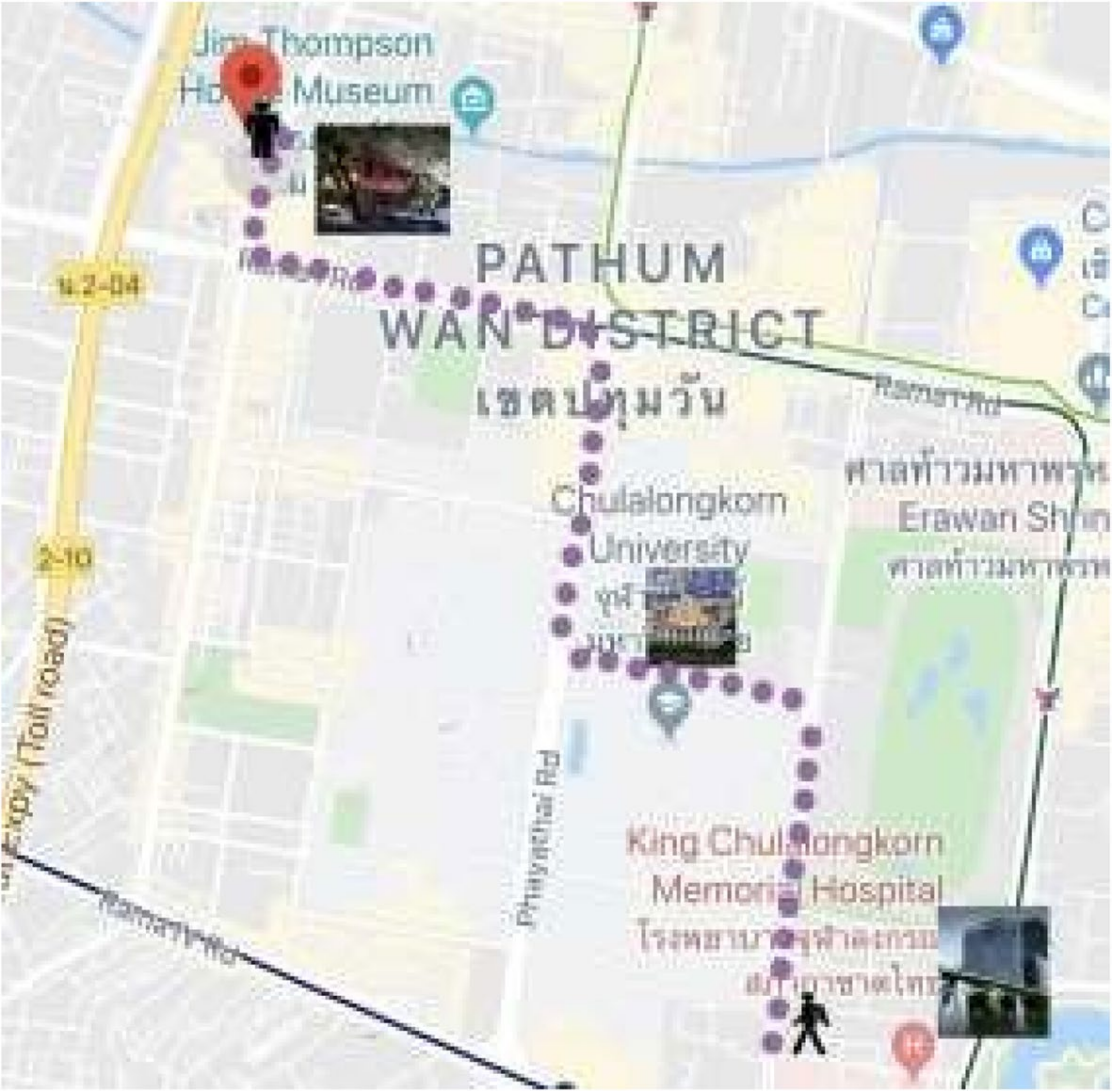
Overview of a stereotypical route that may be navigated by a VI end user in Bangkok, using VIS^4^ION with expanded location microservices

We will recruit a cohort of twenty BLV students to assess the generalizability of our previously described techniques. We will extend our mapping/localization program from the RC campus to a 12-block portion of Bangkok (Fig. 3), giving students the opportunity to more easily access the vibrant city center. To address the challenge of image-based mapping/localization in a large-scale environment, we will divide the large-scale territory to be mapped into smaller territories, roughly breaking down the proposed area of study into one block region. We intend to add a coarse localization tool, using GPS and the embedded IMU in the camera, to efficiently narrow down our search space from the entire territory to a smaller regional city block, subsequently we can deploy our image-based techniques.

### Criteria for discontinuing or modifying allocated interventions {11b}

Principal investigator may terminate or suspend the study under certain circumstances. Circumstances that may warrant termination or suspension include, but are not limited to, unexpected, significant, or unacceptable risk to participants, insufficient compliance to protocol requirements, invaluable or insufficient data, futility determination, and demonstration of efficacy that would warrant stopping.

Participants may withdraw or take back their permission to use and share their health information at any time for this research study. If participants withdraw permission, we will not be able to take back information that has already been used or shared with others.

### Strategies to improve adherence to interventions {11c}

Since subjects will be in the study for a sustained period (for each respective phase), given scheduling requirements, the dedicated research coordinator will clearly communicate expectations to help overcome any potential difficulties. We will make our best attempts to obtain 3 different contact options to ensure that we are able to communicate with the subjects on an ongoing basis.

We will ensure that access to study facilities is aided by route mapping/transportation planning, ensuring the retention of patients given inconvenient geographic logistics from the research site-to-domicile.

Training of clinical and study staff will ensure streamlined communication and avoid miscommunication. A research coordinator at Ratschasuda College will coordinate study assessments while the investigative team will maintain open channels of communication between relevant study facilities for any query at any time point. A clear division of labor and frequent communication will ensure that we retain subjects in the study.

### Relevant concomitant care permitted or prohibited during the trial {11d}

Not applicable. There are no restrictions on concomitant care.

### Provisions for post-trial care {30}

Not applicable. We are not anticipating any post-trial risk.

### Outcomes {12}

The metrics for the entire proposal are detailed as follows:

**Table:** 

Aims	Aim 1	Aim 2	Aim 3	Aim 4	Aim 5
Tasks	1) Multiple correlation of actual to inferred 3D position (goodness of fit metric comparing the actual and inferred 3D positions) 2) Average error as a function of distance from the center of the 3D map (mapping precision will likely vary as a function of position within the mapped region). The precision metric compares performance consistency to the ideal) 3) Positioning (xy) accuracy and viewing angle accuracy (comparing actual performance to the ideal, i.e., accuracy, shows the size and direction of biases in performance) 4) Beta localization	1) Average changes in normalized path length and variability in path length (i.e., percent change in path length and path variance; comparisons of pre/post path length indicate a potential change in confidence and ability in following a path without getting lost) 2) Average changes in normalized traversal time (i.e., percent change in time; comparison of pre/post traversal times indicates potentially improved general mobility and navigation) 3) For destinations that denote social activities, compute mean difference in time spent on such activities pre to post (comparisons between pre/post means indicate potential changes in levels of social interaction) 4) Pre vs. post fall rates (a comparison to determine if fall rates increase/decrease) 5) Assess QoL using the VFQ25 and WHO-QOLBREF 6) Assess physical function and mobility using IPAQ	Measure acceptability, feasibility, and appropriateness as lumped average scores (5-point Likert scale) across post-interview questionnaire and focus-group discussions	1) Average changes in normalized path length (i.e., percent change in path length) 2) Average changes in normalized traversal time (i.e., percent change in time) 3) For destinations that denote social activities, compute mean difference in time spent on such activities pre-to-post 4) Measure overall activity using total path length/day and total travel time/day 5) Measure BP and BMI pre-topost, and correlate with measures 1–4 6) Pre vs. post fall rates 7) Assess QoL using the VFQ25 and WHO-QOLBREF 8) Assess physical function and mobility using IPAQ 9) We will also conduct a process evaluation according to the Saunders Framework	1) Multiple correlation of actual to inferred 3D position (goodness of fit metric) 2) Average error as a function of distance from the center of the 3D map (mapping precision will likely vary as a function of position within the mapped region) 3) Compare (*t*-test) correlations and (linear model) average error functions between Aims 1 and 5 to show generalizability

### Participant timeline {13}

**Table:** 

				**Study period**					
	**Enrolment**	**Allocation**		**Post-allocation**					**Close-out**
**Timepoint (Y/M)**	***Y1/M3***	**0**	***Y1/M9***	***Y2/M3***	***Y2/M12***	***Y4/M6***	***Y5/M6***	***Y5/M9***	***Y5/M12***
**Create and establish DSMB**	X								
**Develop study protocol and SOP for testing and staff training**	X								
**Finalize ICF and recruitment materials/IRB approval**	X								
**Allocation**		X							
**Participant enrollment**	X	X	X	X	X	X	X	X	
**Aim 1**			X						
**Aim 2**				X					
**Aim 3**					X				
**Aim 4**						X			
**Aim 5**								X	
**Concurrent data processing for all Aims**			X	X	X	X	X		
**Preliminary data complete**				X	X	X	X		
**Preliminary data analyses, presentations, manuscripts**				X	X	X	X	X	
**Final statistical analyses, presentations, manuscripts**								X	X

*DSMB* Data Safety Monitoring Board, *SOP* standardized operating procedures, *ICF* informed consent form, *IRB* Institutional Review Board, *M* month

### Sample size [1]

A total of 40 visually impaired subjects will be recruited over the 5 years of this proposed research program. In the first phase, we will focus on demonstrating that travel has improved, using a metric based on the ratio of path length. Path length is a non-normal variate, right-skewed, and must be transformed to satisfy the constraints of a parametric statistical test.

We use log-transformed path-length ratios for the power calculation, by which we determined that, with a statistical power of at least 0.8, we will be able to detect a path-length difference for 20 subjects in which the initial path lengths are 40% shorter than those taken when using the navigation aid, assuming a standard deviation of length-ratios no larger than 2.5.

### Recruitment {15}

We will recruit subjects from Mahidol University’s Ratchasuda College, a school dedicated to those with disabilities. Recruitment will be accomplished through a combination of announcements made at periodic seminars by local study investigators, flyers, and digital e-blasts (emails). All materials will be IRB-approved, and the curation of recruitment content will be overseen by a team of highly trained research coordinators at Mahidol University.

Forty BLV subjects will be recruited over the 5-year research timeline.

We anticipate a subject population of approximately 50% males and 50% females aged 18 and older diagnosed with visual impairment. Subjects, once identified, will be notified of the study using IRB-approved study communication.

A dedicated study research coordinator will perform screening to determine eligibility for the study and schedule the patients for enrollment and subsequent assessments at specified time points.

## Assignment of interventions: allocation

### Sequence generation {16a}

During the study’s second phase, a block randomization will be conducted, assigning participants to either group A or group B. When participants agree to participate, a number will be randomly assigned to them using Excel random number generator. Then, two randomization lists will be created using Excel random number generator, creating separate lists for group A and group B.

### Concealment mechanism {16b}

Participants’ allocated numbers will be created by computer randomization. Sequences will only be revealed when participants are assigned, coded directly into each data file by the computer randomization.

### Implementation {16c}

Designated team member will use the randomized list and allocate participants to groups A or B.

## Assignment of interventions: blinding

### Who will be blinded {17a}

The study will be single-blind. Participants and a designated team member will not be blinded to the intervention. However, study team members who introduce and explain the system to each participant will be blinded and will not know if the participants are part of groups A or B. A blinded analyst will perform statistical analysis.

### Procedure for unblinding if needed {17b}

Team members will be unblinded during the study period only if there are technical systems problems.

Unblinding of the data analyst is not intended.

## Data collection and management

### Plans for assessment and collection of outcomes {18a}

After each session, the collected data will be audited by a designated study team member to ensure the quality of collected data and troubleshoot any unforeseen technical issues. In addition, we will use IMU-based altering to monitor for any falls. For our subjective assessments, we will use the 25-Item National Eye Institute Visual Function Questionnaire (VFQ25) [38], the most frequently used measure of patient-reported, vision-related functioning. The NEI VFQ-25 was developed based on qualitative research with patients to measure the range of vision-related functioning experienced by persons with VI. We will use the International Physical Activity Questionnaire (IPAQ) to assess physical activity. It is a widely used self-reported questionnaire with excellent test reliability for overall score (ICC = 0.81) and physical activity (ICC = 0.84–1.00) [30]. We will administer the World Health Organization QoL Brief (WHO-QoL-BREF) to assess and compare the quality of life. WHO-QoL-BREF is 26 items, self-reported questionnaires that address 4 domains of quality of life, including physical health, psychological health, social relationships, and environments. This test was developed collaboratively and field-tested across several cultural contexts, therefore it is highly recommended to study outcomes and/or measure the quality of life [32, 33].

### Plans to promote participant retention and complete follow-up {18b}

A research coordinator at Ratschasuda College will coordinate study assessments while the investigative team will maintain open channels of communication between relevant study facilities for any query at any time point. A clear division of labor and frequent communication will ensure that we retain subjects in the study.

### Data management {19}

All data management activities will be rigorously and directly supervised and monitored by the PI and Co-PI. All computerized data files will be de-identified and will not contain any information that could be used to identify individual subjects. Rather, data files will be assigned a unique alphanumeric identifier. The code key connecting the data with the subjects’ names will be kept in a separate, secure location. Data will be backed up to a secure research drive. Only the PI and co-investigators will be able to access the data.

### Confidentiality {27}

Confidentiality of research records will be strictly maintained. All computerized data files will be de-identified and will not contain any information that could be used to identify individual subjects. Rather, data files will be assigned a unique alphanumeric identifier. The code key connecting the data with the subjects’ names will be kept in a separate, secure location. Data will be backed up to a secure research drive. Only the PI and coinvestigators will be able to access the data.

The results of these experiments may be published (book or journal) or used for teaching purposes. The identities of participants and participants’ family members will not be revealed in any publication or teaching material without specific permission. After publication, the data may be removed from the PI’s computers and file servers.

The PI will promptly report all adverse reactions to the IRB. An annual report on data and safety monitoring will be submitted to the IRB. This report will detail any adverse events.

### Plans for collection, laboratory evaluation, and storage of biological specimens for genetic or molecular analysis in this trial/future use {33}

Not applicable; the study does not involve biological specimens.

## Statistical methods

### Statistical methods for primary and secondary outcomes {20a}

#### Aim 1

**Fig. 4:**
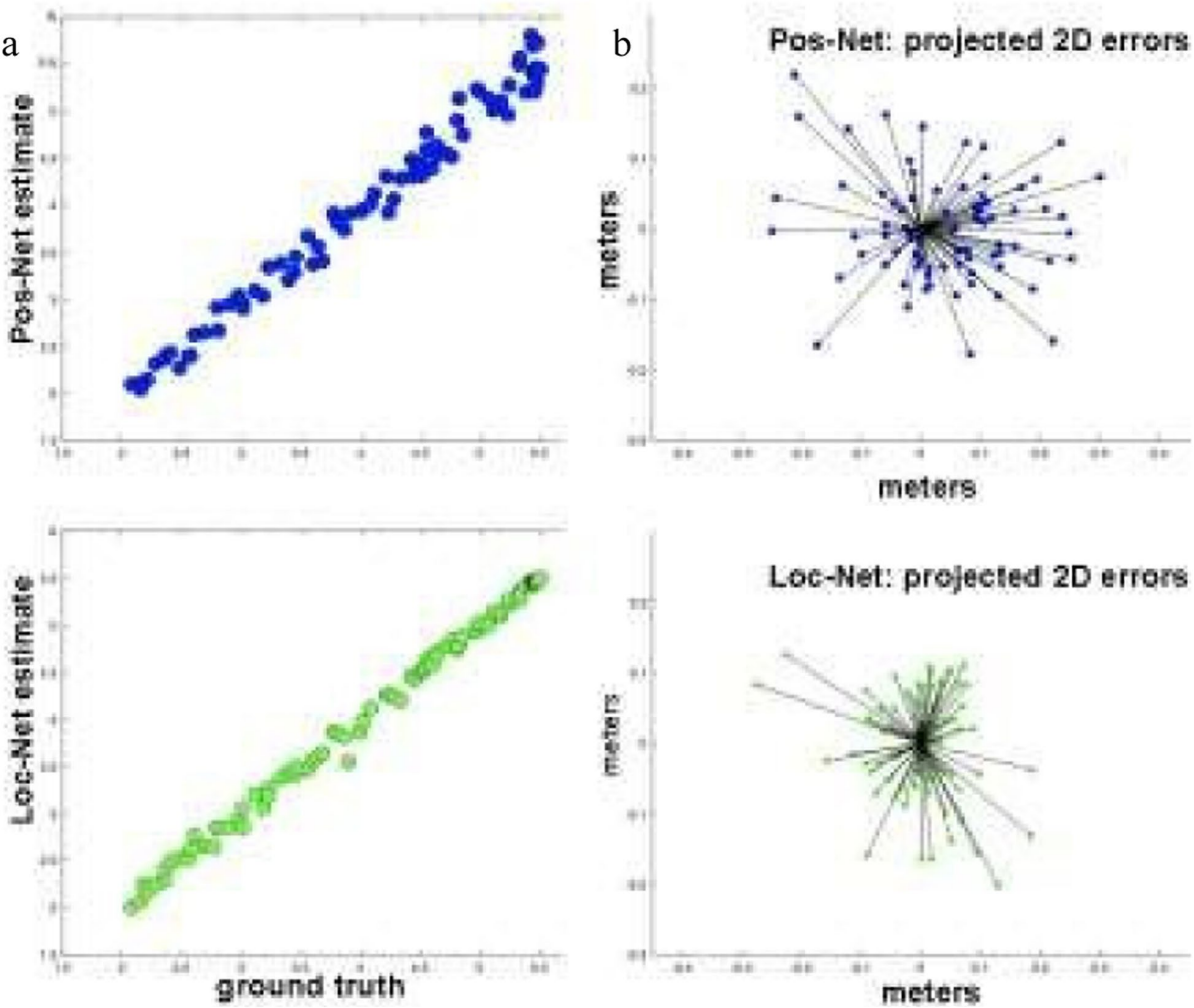
Correlations between ground truth positions and those computed from Pos-Net and Loc-Net (left) and corresponding 2D errors (right)

We expect that mapping based on the mapping microservice (Map-Net) will demonstrate sub-meter level distance accuracy, orientation accuracy of < 5 degrees, and good precision. Our tests of mapping performance will include:

- Multiple correlations of actual to inferred 3D position/orientation (goodness of fit metric), please see Fig. 4a
- Average error as a function of distance from the center of the 3D map, please see Fig. 4b
- Efficiency targets (coverage: 1 block/pedestrian hour of mapping; data storage: 1 block of image data = 200 Mb)

We have collected a small sample of preliminary data (Fig. 4) to demonstrate the feasibility of these analyses, and that they are capable of demonstrating improvement (green; Loc-Net) relative to our baseline (blue; prior art) methods. There is a clear, qualitative improvement in the green plots, both in precision (left, improved correlation) and accuracy (right, error scatter). Our ability to discriminate between methods will only improve with the larger dataset proposed for this aim.

#### Aim 2

We will use the trained network to predict the real-time pose of a BLV user and create a mobility home range for each participant during both the passive and active phases of this initial pilot. We anticipate that route planning will improve through more efficient wayfinding in the active phase, when users have a more comprehensive understanding of the local environment (spatial cognition). We will extract paths between pre-selected pairs of locations during the active and passive phases (e.g., residence to classroom building) traversed in the pre (first 5 days) and post (last 5 days) periods to evaluate our predictions, measuring:

- Average changes and variability in path length (i.e., percent change in path length [difference test based on binomial distribution] and path variance [F-test of variances])
- Average changes in normalized traversal time (i.e., percent change in time [difference test based on binomial)
- For destinations that denote social activities, estimate the difference in time spent on such activities pre to post [time difference based on Wilcoxon signed rank test].
- Pre vs. post fall rates using an exponential waiting-time model of time between falls [Poisson rate of falling]
- Total area covered on an hourly, daily, and weekly basis to assess a home range

#### Aim 3

We will perform a content analysis of the transcripts and notes using NVIVO software [36]. Specific quotations, group interactions, and observations will be assigned codes based on content. The following are the core acceptability and appropriateness outcomes that will be assessed: (a) satisfaction or agreeability with the refined intervention design; (b) perceptions on how the intervention can be adopted within their setting; (c) perceived willingness or interest to participate; and d) perceived relevance and fit of the intervention for the setting. Significant inductive (emerging) codes will also be identified, assessing for differences by sex. Coded items will be grouped into a hierarchical, branching structure in which broad concepts are first identified. The themes and interpretive ideas emerging from those constructs are labeled using constant comparison techniques and subsequently synthesized using matrices structured by the main themes of the analysis [39, 40]. In addition, we will measure acceptability, feasibility, and appropriateness as lumped average scores (5-point Likert scale) across a 27-question post-interview questionnaire. Averages of > 2.5 will indicate an overall positive rating on each dimension, although low average scores on individual questions within each dimension (i.e., non-lumped) will be used to aid product development.

#### Aim 4

We will evaluate the effectiveness of our approach to increase efficient mobility among BLV students, as well as health and well-being. Descriptive statistics will be generated for quantitative data, and thematic content analysis will be performed for qualitative data. We will perform a content analysis of the transcripts and notes using NVIVO software [36], in a manner similar to what has been described above in Aim 3. Results will be categorized according to the seven dimensions of the Saunders Framework: fidelity, dose delivered, dose received (exposure), dose received (satisfaction), recruitment, reach, and context. Formative data will be reported iteratively to the study team throughout the trial, in order to improve intervention delivery, while summary data will be reported at the end of the trial along with the results of the trial. We will compare pre/post-differences in subjective assessments and in navigation and health metrics.

**Fig. 5:**
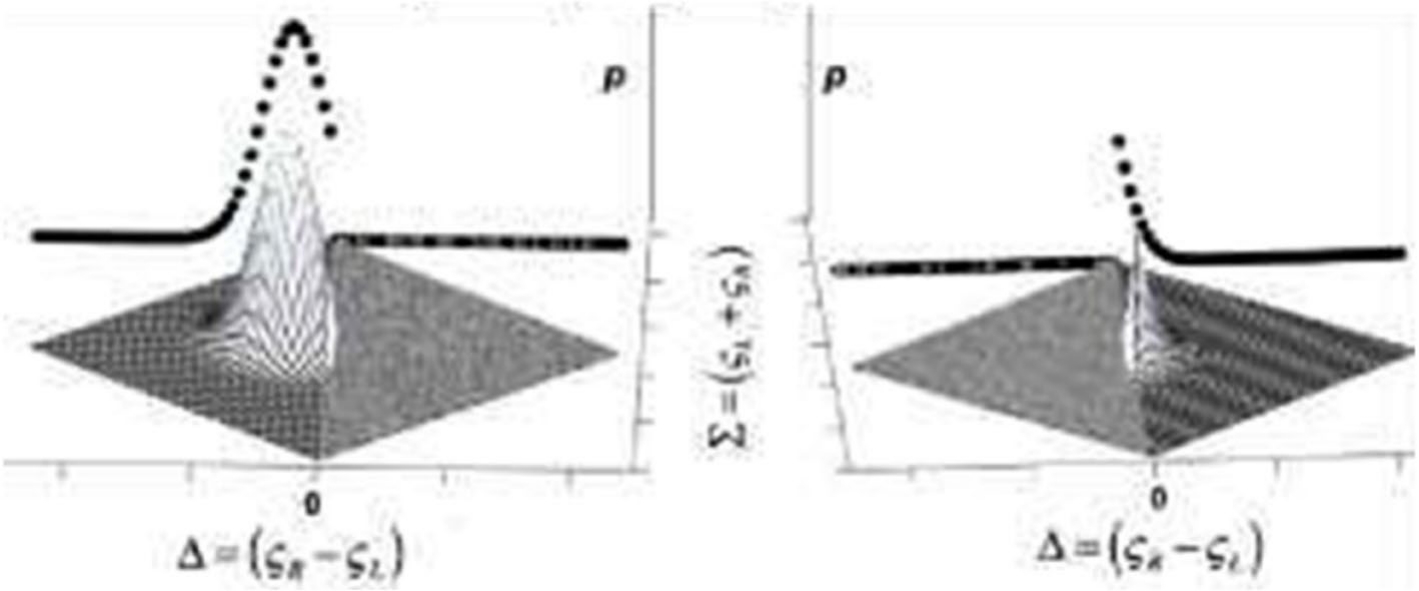
2D posterior distributions over differences ($\Delta$) and sums ($\Sigma$) of the two poisson rate parameters ($\varsigma$), separated along the $\Delta =0$ axis. Marginalizing over this 2D posterior either along the $\Delta =0$ axis $\left({M}_{\Delta =0}\right)$, or for all portions of the posterior that exclude the $\Delta =0$ axis $\left({M}_{\Delta \ne 0}\right)$, underlie the model comparison between the equal rates $\left({M}_{\Delta =0}\right)$ and unequal-rate $\left({M}_{\Delta \ne 0}\right)$ models

We will compare pre-post metrics using Bayesian model comparison techniques. For example, we will compare fall rates using an exponential waiting-time model of duration between falls (Poisson rate of falling,) based on the ratio (Fig. 5):

$$p \left(S \right|\varsigma )={e}^{-\varsigma \left(\Delta t\right) }\frac{\varsigma \left(\Delta t\right)}{{S}^{t}} \frac{p \left({M}_{\Delta =0 }\right|data\bullet t)}{p \left({M}_{\Delta \ne 0 }\right|data\bullet t)}\propto \frac{\int d\varsigma p \left({S}_{1}\bullet {S}_{2}\right|{\varsigma }_{1}={\varsigma }_{2})}{\iint d{\varsigma }_{1}d{\varsigma }_{2}p \left({S}_{1}\bullet {S}_{2}\right|{\varsigma }_{1}\ne {\varsigma }_{2})}$$

#### Aim 5

This generalization experiment is expected to continue to produce sub-meter accuracy and precision for the localization of participants using the VIS^4^ION platform. We will assess this prediction, as well as directly compare localization metrics obtained with this experiment to those from prior Aims 1, 2, and 4.

- Multiple correlations of actual to inferred 3D position (goodness of fit metric)
- Average error as a function of distance from the center of the 3D map
- Compare correlations (via *t*-test) and average error functions (via linear model) between Aims

We will adopt similar analyses, as explained in prior Aims, to access localization accuracy. In addition, we will add GPS and IMU signals (not used earlier) to detect falls/collisions and localize these injuries to determine the potential for safety alerts associated with particular types of terrain and building or landscape structures.

### Interim analyses {21b}

Weekly data quality checks by TH and JR will also be used to monitor that collisions have not occurred during the testing phase beyond the rate seen during baseline data collection.

$$O \left({M}_{i}\right)=\frac{p \left({M}_{i}\right|d\bullet t) }{p \left({\overline{M} }_{i}\right|d\bullet t)}= \frac{{\varpi }_{i}{\mathcal{L}}_{i}}{{\sum }_{j\ne i}{\varpi }_{j}{\mathcal{L}}_{i}}$$

$$\mathcal{L}\left(\zeta \right)=p\left(d | \zeta \bullet {M}_{2}\bullet tp\right)= {\prod }_{i}\frac{{e}^{-\zeta {d}_{i}}}{{d}_{i}!}\propto {e}^{-\zeta }{\prod }_{i}\zeta {d}_{i}$$

A Bayesian model selection [41] in which a Poisson model of occurrence rate comparing the hypotheses that the rate is less than or equal to the baseline vs. the hypothesis that the rate is greater during testing will be used to assess whether the trial should be halted early. PI will make the final determination.

### Methods for additional analyses (e.g., subgroup analyses) {20b}

Post hoc analyses will be treated as exploratory hypothesis testing and labeled as such in any written reports. Such analyses will allow us to explore additional potential connections found within our data, and plan for future studies to formally test such hypotheses. No post hoc analysis will be used to form the basis for general conclusions or recommendations issued from these studies.

### Methods in analysis to handle protocol non-adherence and any statistical methods to handle missing data {20c}

Missing data are expected to be distributed according to MAR [41]. If the rate of missing data (r) is not related to the multivariate output (y) after taking account of x (inputs):

## $P\left(r_i=0\vert{\mathcal x}_{i,}{\mathcal Y}_{mis,\mathfrak i;}\phi\right)=P\left(r_i=0\vert{\mathcal x}_i,;\phi\right),$

Then the missing data are ignorable. However, if instead, this simplification is not valid, such that NMAR holds, then the data are not ignorable, and a Bayesian multiple imputation technique based on the prior predictive distribution (Hudson, 2021) or a sampling-based technique for generating samples of data (McElreath, 2016) meeting the characteristics of the missing data will be used to account for missing data.

### Plans to give access to the full protocol, participant-level data and statistical code {31c}

We will create a data archive that will contain raw study data (no identifiers). These data will be available to other investigators upon formal request to the principal investigator, following the publication of the primary analyses of the project, and after the data have been appropriately checked, cleaned, and de-identified. The archive will reside on a server for which appropriate firewalls and other forms of data protection will be installed for maximum security. Prospective users will complete an application for use of the data, which will include queries about the users’ project hypotheses and proposed analyses. All NIH regulations and restrictions as well as HIPAA Security Rules regarding personal health information will be observed.

## Oversight and monitoring

### Composition of the coordinating center and trial steering committee {5d}

*NYU Grossman School of Medicine*: this will be the primary site for general management and overview of the study, as well as statistical analysis, and formulation of presentations and manuscripts. *Mahidol University*: The Ratchasuda College and Faculty of Information and Communication Technology (ICT) will contribute to this research project by mapping areas of interest and conducting the field trials of VIS^4^ION with visually impaired students at Ratchasuda College, Mahidol University, Salaya campus and within the extended environment in urban Bangkok, as well as the investigations focused on human and system adoption in different contexts, assessing the impact on quality of life in a low-resource setting and gauging future scalability. ICT members will integrate required hardware and software for study experimentation purposes.

*NYU Tandon*: School of Engineering: a primary site for neural network data analysis and developing the wearable device.

### Composition of the data monitoring committee, its role and reporting structure {21a}

Data Monitoring Committee (DMC): is the primary body for study-related decision-making and is responsible for its successful completion, including target enrollment, subject retention, and protocol adherence. The Committee comprises all study investigators. PI is the chair of the Committee. On issues requiring a vote, one vote per member will be allowed. The ultimate authority over the conduct of the research will reside with the study PI. The DMC meets monthly or more frequently, as required.

The primary responsibilities of the DMC include:

- + Approving all study-related documents, including:
- Study protocol,
- Informed consent form, and
- Data and safety monitoring plan (DSMP),
- + Reviewing and subsequently voting on top protocol and operational issues;
- + Receiving and reviewing all reports from team members, in order to provide recommendations and guidance regarding potential study issues;
- + Assisting in the development of corrective action plans if enrollment targets are not being met or noncompliance with the protocol has been noted; and
- + Overseeing the dissemination and publication of study results.

### Adverse event reporting and harms {22}

Overall, the risk to participants is minimal: the likelihood of physical/psychological harm does not exceed what would be encountered in ordinary life. Subjects will be learning how to use a new assistive device and during this learning period, the subjects may be at a slightly greater risk of injury. Once the subject is accustomed to the device, the risk of injury would not exceed that in ordinary life.

The proposed psychophysical procedures pose no risk (the most likely negative consequence being boredom or fatigue). Subjects will be encouraged to rest should they feel any fatigue.

This risk is minimized through careful handling and storage of data and training of staff members. Subjects will be told to contact the PI if they experience a research-related injury. Should new information become available during the research that may affect a participant’s willingness to participate, all participants will be notified. Additionally, should any adverse effects of the procedures be demonstrated in the future, every attempt to notify participants will be made.

All adverse events will be immediately reported to the Ethics Committee.

### Frequency and plans for auditing trial conduct {23}

We have bi-weekly meetings to assess research completion as set in the research outline and weekly data quality monitoring. PI and Co-Is receive monthly reports from team members during recruitment and experiment to monitor adherence to protocol. We will assess the dissemination of activities by tracking peer-reviewed publications, presentations in national and international conferences, and poster presentations.

We submit NIH’s annual progress report. Also, the Independent Monitoring Committee (IMC) audits the trial. The IMC will comprise three members, a PhD-level biostatistician and two experts in the field. However, they are not otherwise associated with this research project and work independently of the PI. The IMC will meet annually and recommend to the PI whether the study should continue as planned, continue with modifications, or be stopped.

### Plans for communicating important protocol amendments to relevant parties (e.g., trial participants, ethical committees) {25}

The reportable events noted above will be reported to the IRB using the form: “Reportable Event Form” or as a written report of the event (including a description of the event with information regarding its fulfillment of the above criteria, follow-up/resolution and need for revision to consent form and/or other study documentation). Copies of each report and documentation of IRB notification and receipt will be kept in the Clinical Investigator’s study file.

### Dissemination plans {31a}

The final data will be made available in formats that are considered an industry standard for our field of research. The data elements will enable comparisons between research studies and allow for reproducibility, facilitating larger research endeavors. All data will be de-identified; anonymized forms will be disseminated. Publication of the data sets relevant to this study proposal will follow the publication of research findings from the study-specific projects. Data will also be used for national meetings and symposia.

The results of these experiments may be published (book or journal) or used for teaching purposes. The identities of participants and participants’ family members will not be revealed in any publication or teaching material without specific permission.

## Discussion

Perhaps the greatest difficulties and dangers faced by the BLV involve those encountered during locomotion and navigation; ameliorating these difficulties would significantly improve the lives of these individuals. Although electronic aids for navigation seem like an attractive solution, there are several barriers to their use, chief among them being their dependence on either environmental (sensor-based) infrastructure or WiFi/cellular “connectivity” infrastructure or both. Here we propose a navigation solution that operates independently of both environmental and Wi-F/cellular infrastructure, as well as tests of both the performance of the system and the function/health benefits gained from use. Furthermore, this system was uniquely created to excel in low-resource settings with poor telecommunications. Thus, it is uniquely poised to deploy in LMICs. In the current proposal, we intend to deploy the system and perform staged evaluations within environments of progressive difficulty; we also intend to acquire patient-centered feedback from interviews and questionnaires.

Future studies are needed to promote the expansion of software-based mapping and localization microservices deployed through advanced wearables. More broadly, advanced wearables may serve as a foundation on which to build modular wayfinding tools turned into best practices for improved health and well-being. While our current proposal helps to build upon rigorous prior literature and expand our knowledge base regarding assistive technology and implementation science in low-resource settings for the BLV, we intend to expand the disabilities addressed and experiment with both cognitive and social disabilities, including mild cognitive loss, dementia, and autism. As the study of BLV ecology (mobility phenotyping [42]) is a robust and logical next step in the science of wayfinding, we intend to further diversify the disabilities characterized, geographic regions examined, and cultural contexts incorporated.

## Trial status

The study has not yet started enrolling participants; the anticipated start date is 2023.02.20, and the anticipated termination date is 2026.10.01.

## Data Availability

All computerized data files will be de-identified and will not contain any information that could be used to identify individual subjects. Rather, data files will be assigned a unique alphanumeric identifier. The code key connecting the data with the subjects’ names will be kept in a separate, secure location. Data will be backed up to a secure research drive. Only the PI and co-investigators will be able to access the data.

## Abbreviations

LMIC: Low- and middle-income countries
RC: Ratchasuda College
VIS^4^ION: Visually Impaired Smart Service System for Spatial Intelligence and Onboard Navigation
QoL: Quality of life
IPAQ: International Physical Activity Questionnaire
WHO-QoL-BREF: World Health Organization Quality of Life Brief
BLV: Blind and visually impaired
3D: Three-dimensional
2D: Two-dimensional
MU: Mahidol University
ETA: Electronic Travel Aids
FPS: Frame per second
REACTIV: Rehabilitation and Engineering Alliance & Center Transforming Impaired Vision
NYU: New York University
IMU: Inertial Measurement Unit
SfM: Semantic segmentation and structure from motion
6DOF: 6 Degrees-of-freedom
SLAM: Simultaneous localization and mapping
GPS: Global positioning system
NYC: New York City
FGD: Focus group discussion
VFQ25: 25-Item National Eye Institute Visual Function Questionnaire NIHNational Institutes of Health
RE-AIM: Reach, Effectiveness, Adoption, Implementation, Maintenance PRISMPerformance of Routine Information System Management
HIPAA: Health Insurance Portability and Accountability Act
